# Comparing Early Intervention to Watchful Waiting: A Review on Risk Stratification and Management in Asymptomatic Aortic Stenosis

**DOI:** 10.3390/medicina61030448

**Published:** 2025-03-04

**Authors:** Ahmed E. Khedr, Nour B. Odeh, George Bcharah, Hesham M. Abdalla, Abdulrahman Senjab, Rawan M. Zeineddine, Jaikrishnan Ram, Juan M. Farina, Owen R. Crystal, Bryan Barrus, Steven J. Lester, Justin Shipman, Said Alsidawi, Chadi Ayoub, Kristen A. Sell-Dottin, Reza Arsanjani

**Affiliations:** 1 Department of Cardiothoracic Surgery, Mayo Clinic, Phoenix, AZ 85054, USA; khedr.ahmed@mayo.edu (A.E.K.); odeh.nour@mayo.edu (N.B.O.); senjab.abdulrahman@mayo.edu (A.S.); zeineddine.rawan@mayo.edu (R.M.Z.); farina.juanmaria@mayo.edu (J.M.F.); barrus.bryan@mayo.edu (B.B.); sell-dottin.kristen@mayo.edu (K.A.S.-D.); 2 Department of Cardiothoracic Surgery, Mayo Clinic Alix School of Medicine, Phoenix, AZ 85054, USA; bcharah.george@mayo.edu (G.B.); ram.jaikrishnan@mayo.edu (J.R.); 3 Department of Internal Medicine, Mayo Clinic, Phoenix, AZ 85054, USA; abdalla.hesham@mayo.edu; 4 Department of Cardiovascular Medicine, Mayo Clinic, Phoenix, AZ 85054, USA; crystal.owen@mayo.edu (O.R.C.); lester.steven@mayo.edu (S.J.L.); shipman.justin@mayo.edu (J.S.); alsidawi.said@mayo.edu (S.A.); ayoub.chadi@mayo.edu (C.A.)

**Keywords:** asymptomatic aortic stenosis, risk stratification, clinical surveillance, aortic valve replacement, clinical guidelines, intervention

## Abstract

Aortic stenosis is a progressive condition with substantial implications for morbidity and mortality. In recent years, attention has shifted toward risk stratification and the development of individualized management plans to optimize treatment outcomes. The management of asymptomatic patients has become a topic of significant controversy, as emerging studies challenge traditional watchful waiting guidelines and propose the potential benefits of early intervention. While early intervention may reduce overall morbidity and mortality in this patient population, the associated procedural risks remain a critical consideration. This review seeks to analyze the existing literature, offering an updated perspective on patient risk stratification and evidence evaluating both management approaches.

## 1. Introduction

Aortic stenosis (AS) is the most common valvular heart disease, characterized by the narrowing or restriction of the aortic valve opening, resulting in turbulent blood flow with increased velocities as it transits from the left ventricle (LV) to the aorta [1,2]. Its prevalence increases significantly with age, impacting up to 10% of individuals by the eighth decade of life [1,3]. With an aging population, the burden of AS is expected to rise further, emphasizing the need for enhanced screening, timely diagnosis, and advancements in treatment options to address this growing public health challenge.

Patients with severe asymptomatic AS are typically managed with active surveillance, which includes regular clinical assessments and echocardiographic monitoring. However, patients with severe asymptomatic AS frequently progress to the development of symptoms, with studies indicating that a significant proportion experience angina, dyspnea, or syncope within a few years [4,5]. The evolving landscape of AS management has sparked ongoing debate around shifting the inflection point for earlier intervention for patients with severe asymptomatic disease. This shift is largely driven by advancements in surgical outcomes and the widespread availability of transcatheter aortic valve replacement (TAVR) [6,7]. These improvements have reduced procedural risks and mortality, prompting reconsideration of the traditional “watchful waiting” strategies. Advocates for earlier intervention highlight the potential to preempt symptom development and adverse cardiac remodeling, while skeptics emphasize the need for precise risk stratification to avoid overtreatment. This debate underscores the critical balance between leveraging technological progress and ensuring individualized patient care. This review explores the existing literature and evaluates the relative merits of active surveillance vs. early intervention in patients with severe but asymptomatic AS.

## 2. The Critical Role of Timely Intervention

Hemodynamically severe AS is defined by an aortic velocity ≥4 m/s or a mean transvalvular pressure gradient ≥40 mm Hg. Aortic valve area (AVA) is typically ≤1.0 cm^2^ although this is not a required criterion. Asymptomatic patients with severe valvular obstruction are classified as having stage C disease. Stage C is further subdivided into C1 and C2 based on left ventricular (LV) systolic function, with a left ventricular ejection fraction (LVEF) <50% signifying Stage C2 [4].

**Figure 1:**
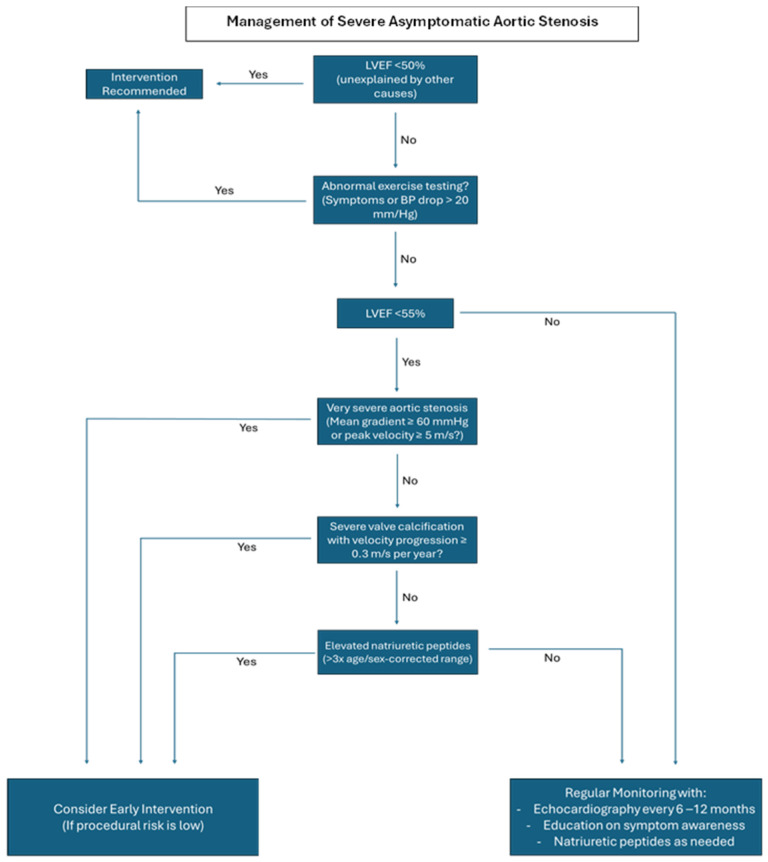
Algorithm for the management of patients with severe asymptomatic aortic stenosis, outlining key decision points for monitoring and intervention, according to the ESC/EACTS guidelines.

Guidelines from both the European Society of Cardiology (ESC)/European Association for Cardiothoracic Surgery (EACTS) and the American College of Cardiology (ACC)/American Heart Association (AHA) provide little controversy on the timing of intervention for patients with either asymptomatic severe AS and a reduced LVEF (stage C2) or symptomatic severe AS (stage D) providing a class 1 indication for valve intervention [4,8]. Figure 1 illustrates the current ESC/EACTS guidelines for the management of severe aortic stenosis.

Patients with severe AS who remain truly asymptomatic still face an approximately 50% higher risk of all-cause mortality, as well as an increased risk of acute myocardial infarction, stroke, and heart failure hospitalizations, compared to those undergoing early surgical intervention [9]. Additionally, within 5 years of diagnosis, approximately two-thirds of conservatively managed patients with asymptomatic severe AS will develop symptoms, and 75% will have either died or undergone aortic valve replacement (AVR) [10]. Based on this understanding along with significant reductions in periprocedural mortality for surgical aortic valve replacement (SAVR) and transfemoral TAVR, the debate between active surveillance and timely intervention for some stage C1 patients continues.

For patients undergoing intervention, the choice between SAVR and TAVR is influenced by several factors, including cost-effectiveness, durability, and patient preferences. Studies comparing procedural and long-term costs have found that transfemoral TAVR is generally more cost-effective than SAVR, making it an economically favorable option [11,12]. However, concerns remain regarding TAVR’s long-term durability. A 2020 study comparing SAVR with two generations of the SAPIEN TAVR device found that the 5-year Kaplan–Meier cumulative rate of SVD was significantly higher in the SAPIEN XT group than in the SAVR group (9.5% [95% CI: 7.0–12.7%] vs. 3.5% [95% CI: 2.1–5.8%], *p* < 0.001), whereas the SAPIEN 3 demonstrated comparable SVD rates to SAVR (3.9% [95% CI: 2.5–6.0%] vs. 3.5% [95% CI: 2.1–5.8%], *p* = 0.65) [13,14]. Beyond clinical outcomes, patient preferences also play a crucial role in decision making. Studies have shown that greater patient involvement in the selection process leads to improved outcomes, with many patients prioritizing TAVR due to its lower short-term mortality and less invasive nature [15,16,17].

## 3. Risk Stratification and Diagnosis

Risk stratification and diagnostic evaluation are the cornerstones of managing asymptomatic AS, helping to identify patients who may benefit from closer monitoring or earlier intervention. Evaluation through cardiovascular imaging, exercise testing, and cardiac biomarkers allows for the identification of high-risk patients prone to rapid progression or adverse outcomes.

### 3.1. Echocardiography

**Figure 2:**
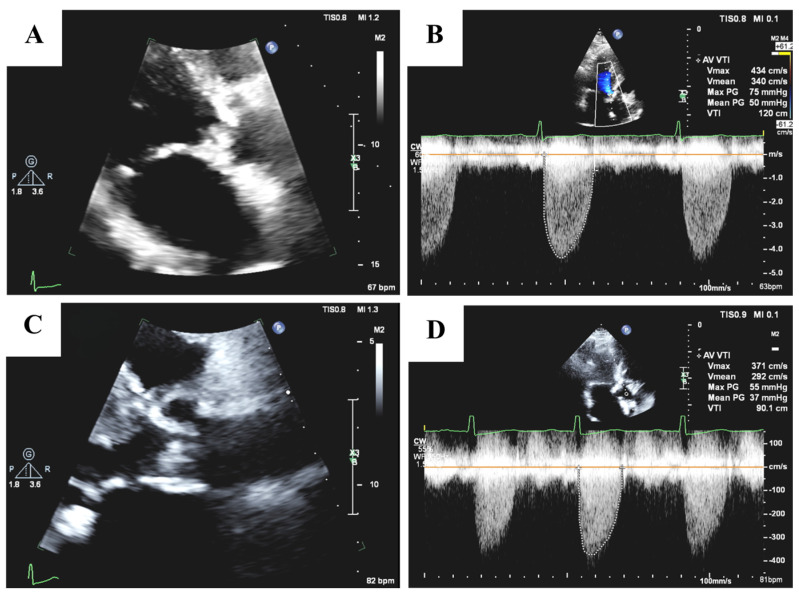
(**A**) Echocardiography image from a parasternal view focused on the aortic valve showing severe calcification and narrowing of the aortic valve opening. (**B**) Corresponding echocardiogram with key parameters, including a peak aortic jet velocity of 4.3 m/s and a mean transvalvular pressure gradient of 50 mmHg, indicating severe disease. (**C**) Echocardiography image from a parasternal view focused on the aortic valve showing moderate calcified aortic stenosis. (**D**) Corresponding echocardiogram with key parameters, including a peak aortic jet velocity of 3.7 m/s and a mean transvalvular pressure gradient of 37 mmHg, indicating moderate disease.

Echocardiography is the cornerstone for diagnosing AS and providing comprehensive evaluation of valve anatomy, function, and hemodynamics. Key parameters include peak aortic jet velocity (>4 m/s), mean transvalvular pressure gradient (≥40 mm Hg), and AVA (<1.0 cm^2^), which are critical for grading disease severity, as shown in Figure 2 [18,19,20,21]. LVEF below 50% signify LV dysfunction and worsened prognosis. Stroke volume index (SVI) and valvuloarterial impendence (Zva), also provide additional insights into cardiac function and systemic hemodynamic burden [18,20].

SVI is a simple echocardiographic measure emerging as a tool for staging and risk stratifying AS. Integration of SVI can improve outcome predictions, particularly in cases with low-flow states (SVI < 35 L/m^2^) that may mask true AS severity. Notably, low flow rates have been independently associated with all-cause mortality and a higher risk of hospitalization for heart failure in patients with severe AS [20].

Global longitudinal peak systolic strain (GLS) is emerging as a valuable parameter for guiding the timing of valve intervention in patients with aortic stenosis. Unlike LVEF, GLS provides a more sensitive measure of subtle myocardial dysfunction, often detecting early changes in ventricular performance before LVEF declines [22]. A retrospective study of 220 patients revealed that, despite comparable LVEF, LV-GLS was significantly impaired in patients with asymptomatic severe AS compared to controls. Moreover, patients with impaired LV-GLS had a higher risk of developing symptoms and requiring AVR during follow-up, compared to those with more preserved LV-GLS. This can in turn lead to identifying patients who may benefit from AVR [23]. Overall, reduced GLS has been associated with worse outcomes, including increased mortality and heart failure risk, in patients with severe AS. Its incorporation into clinical practice offers the potential for earlier identification of high-risk patients who may benefit from timely valve replacement, though further research is needed to standardize its use and integrate it into current guidelines.

### 3.2. Cardiac Computed Tomography

Cardiac computed tomography (CCT) plays a crucial role in risk stratification and diagnosis of AS, especially in cases where echocardiography does not yield definitive results [24]. Although echocardiography is the mainstay imaging modality for assessing AS, in up to 40% of cases, findings might be inconclusive [25]. This is when CCT, particularly aortic valve calcium scoring (CT-AVC), may be helpful; as it provides a quantitative, flow-independent measure of AVC with good prognostic capabilities [26,27].

In a multicenter study involving 794 patients, the AVC load effect on overall mortality was evaluated in AS patients undergoing conservative management. They concluded that AVC load is a significant predictor of survival, independent of clinical and echocardiographic characteristics. They further suggested that AVC load (severe AVC defined as ≥1274 AU in women and ≥2065 AU in men) and AVC density (severity defined as more than 292 and 476 AU/cm2 in women and men, respectively) both are important metrics for assessing prognosis in AS patients [28].

Advanced CT modalities like CT angiography (CTA) can help with morphological assessment of the aortic valve, and determination of comorbidities like coronary artery disease, enabling the identification of severe AS and monitoring of disease progression to further guide therapeutic interventions [24]. In a recent study, that included 136 patients with AS, the fibrocalcific volume was calculated on CTA at the baseline and after 1 year. Baseline fibrocalcific volume was a strong predictor of gradient progression, highlighting that this CTA-derived measurement can predict disease progression [29].

A recent study highlighted the emergence of Positron Emission Tomography (PET)/CT in AS evaluation, modalities that can detect inflammation and calcification by utilizing tracers like 18F-FDG and 18F-NaF. The study found that 18F-NaF uptake correlates with AS severity and progression, making it a powerful tool for early diagnosis and risk stratification [30].

### 3.3. Cardiac Magnetic Resonance Imaging

Cardiac Magnetic Resonance Imaging (CMR) offers valuable insights into myocardial structure and function and may play a role in assessing asymptomatic AS patients [31]. CMR helps identify maladaptive remodeling patterns, even in the absence of clinical symptoms and may aid in determining the timing of intervention [32]. Late gadolinium enhancement (LGE) provides a sensitive measure of myocardial fibrosis, which has been linked to poor outcomes such as heart failure and increased mortality in AS even after AVR (qualitative grading of LGE: 0 = no enhancement, 1 = mild insertion point enhancement, 2 = subtle enhancement in one region outside insertion point, 3 = bright scar in one region/diffuse enhancement in multiple regions, and 4 = clear scar in various regions) [33]. For instance, following the PRIMID-AS multicenter, a prospective observational study that involved 170 asymptomatic patients with moderate to severe AS, the 43 patients who were asymptomatic at 12 months had a repeat CMR. This prospective study demonstrated that the LGE burden nearly doubled over 12 months in asymptomatic AS patients, indicating significant progression of cardiac remodeling and the potential need for earlier intervention [33]. CMR has also been compared with exercise testing, as discussed below. Studies indicate that while myocardial perfusion reserve is associated with symptom onset, its predictive accuracy in CMR remains moderate when compared to exercise test results [34,35].

### 3.4. Cardiopulmonary Stress Testing

Cardiopulmonary stress testing (CPET) is emerging as a valuable tool for further risk stratification in patients with asymptomatic severe AS. It provides a comprehensive evaluation of the cardiorespiratory system by measuring parameters such as oxygen consumption (VO_2_), carbon dioxide production (VCO_2_), and ventilatory efficiency. A retrospective study assessed 25 asymptomatic severe AS patients using CPET. The parameters evaluated included the mean minute ventilation/carbon dioxide production slope (VE/VCO_2_), VO_2_ value in the first ventilatory threshold (VT1), peak circulatory power, and oxygen uptake efficiency slope (OUES). OUES was shown to accurately identify a higher degree of functional limitation in patients with submaximal CPET, but its utilization as a prognostic tool has yet to be clinically proven [36]. In a study assessing the prognostic usefulness of CPET (n = 101) in AS, a normal CPET correlated to a good outcome in an initial conservative management with no unexpected cardiac deaths [37].

In a retrospective study of 504 consecutive patients with severe AS (0.6 cm^2^/m^2^ on resting echocardiogram) and preserved LVEF (≥50%), treadmill stress echocardiogram was performed to confirm asymptomatic status where clinical and exercise variables (% of age–sex–predicted metabolic equivalents [%AGP-METs]) were recorded. Despite being asymptomatic, only 252 (50%) of the patients attained >100% AGP-METS suggesting that being asymptomatic does not necessarily mean watchful waiting is the correct approach [38]. Attention to markers such as Zva, as mentioned earlier, in concurrence with evaluating functional capacity in asymptomatic AS may also aid in achieving optimal AVR timing [19].

The role of CPET, stress echocardiography, and Zva in the risk stratification of patients with asymptomatic severe aortic stenosis is evolving. These advanced diagnostic tools offer insights into subclinical dysfunction, hemodynamic burden, and cardiac reserve, providing a more nuanced approach to identifying high-risk patients. However, while promising, the parameters derived from these modalities have not yet been incorporated into current clinical guidelines. Further large-scale studies are needed to validate their prognostic value and define standardized thresholds for intervention, ensuring these tools can be effectively integrated into clinical decision-making frameworks.

### 3.5. Exercise Testing

The evaluation of symptoms can be challenging partly due to their frequent manifestation in a variety of cardiac and non-cardiac conditions, including physical deconditioning, lung disease, and ischemic heart disease. In addition, individuals may subconsciously restrict their activity to avoid triggering symptoms, often rationalizing these changes as the effects of aging or personal preference [39]. Exercise tolerance testing (ETT), previously contraindicated in AS patients now plays a crucial role in the risk stratification of patients who subjectively report being asymptomatic, as a means of unmasking symptoms [40]. A positive ETT is defined as the occurrence of significant symptoms at a less than expected workload, a systolic blood pressure drop exceeding 20 mmHg from baseline, or the presence of sustained tachyarrhythmias. ST-segment depression, known to lack specificity in AS, is often excluded as a criterion for test positivity in this specific scenario [41].

In a prospective study of 66 patients with isolated severe asymptomatic AS, a positive ETT was associated with a seven-fold increase in the risk of symptom development or sudden death [21,42]. A retrospective study involving 316 asymptomatic patients who underwent 797 ETTs found that 52% of those initially asymptomatic developed symptoms during testing, and 30% died during follow-up, predominantly from cardiovascular causes [41]. Specific symptoms observed during ETT—such as exertional dyspnea, angina, syncope, and presyncope—are indicative of AS progression. Patients who exhibit such symptoms on ETT are reclassified as having stage D disease and as such should be promptly referred for AVR [43,44]. Poor exercise tolerance or an abnormal blood pressure response to exercise can indicate higher risk and prompt earlier consideration for intervention with current guidelines providing a class IIa indication for valve replacement in these patients [4].

### 3.6. Biomarkers

Several biomarkers have been investigated for their utility in risk stratification of patients with stage C1 disease. Among these, N-terminal pro-b-type natriuretic peptide (NT-proBNP), a key marker of myocardial stress and dysfunction has demonstrated significant prognostic value. Elevated NT-proBNP levels have been consistently associated with worse outcomes, even in asymptomatic patients.

A prospective study involving 69 patients indicated that serial measurements of B-type Natriuretic Peptide (BNP) could effectively predict the necessity for AV intervention [45]. Similarly, a study of 130 patients, followed over 6 to 9 months, found that asymptomatic patients with severe AS and plasma BNP levels below 130 pg/mL rarely developed symptoms [46]. Another cohort of 70 patients revealed a BNP level of 300 pg/mL or greater as a poor prognostic factor in both symptomatic and asymptomatic patients managed medically [47]. More recently, a prospective study of 1953 consecutive patients with moderate to severe AS reported that those with elevated BNP levels adjusted for age and sex had a significantly higher mortality risk (HR 2.35; [95% CI: 1.57–3.56]) [48].

Other biomarkers, including troponin and Galectin-3, have also demonstrated prognostic significance. Troponin elevation reflects myocardial injury, while elevated Galectin-3 reflects adverse cardiac remodeling and fibrosis [45,49,50]. White et al. conducted a systematic review and meta-analysis with pooled analysis revealing that all-cause mortality in AS was significantly associated with higher levels of BNP (HR 2.59, [95% CI 1.95–3.44], *p* < 0.00001), NT-proBNP (HR 1.73, [95% CI 1.45–2.06], *p* = 0.00001), Troponin (HR 1.65, [95% CI 1.31–2.07], *p* < 0.0001) and Galectin-3 (HR 1.82, [95% CI 1.27–2.61], *p* < 0.001) [51].

This understanding of biomarker profiles provides valuable insight into disease progression, aiding in the timely identification of patients who may benefit from early intervention. This understanding is reflected in current guidelines which provide a class IIa indication for valve intervention in patients with stage C1 disease and a BNP > 3x normal [4].

## 4. Watchful Waiting: Is It Sufficient?

Current guidelines for the management of AS provide a valuable framework for determining when to pursue valve replacement. Guidelines still suggest an approach of active surveillance in many patients with asymptomatic severe AS. The 2020 ACC/AHA guidelines are supported by the decision-analytic model by Gada et al. [52]. They demonstrated that watchful waiting yielded higher quality-adjusted life years (7.4 vs. 5.3 QALYs), and lower costs compared to immediate AVR unless the preoperative risk of sudden death or post-operative heart failure was substantially greater than current estimates (e.g., >13% for annual mortality risk). Such findings reinforce the notion that an initial non-interventional approach can optimize both the quality of life and resource utilization in asymptomatic patients.

Additional support for this paradigm is provided by San Román et al. and Izumi, who highlight the relatively low annual risk of sudden death in asymptomatic severe AS—ranging from about 0.25% to 1.7%—and emphasize the value of careful patient monitoring and education rather than preemptive surgery [53,54]. Ennezat et al., Ledwoch, and Thiele also underscore that careful follow-up, including periodic echocardiography, exercise testing, and biomarker assessment, can allow clinicians to detect subtle changes in valve hemodynamics or early symptom development that may warrant re-evaluation of their management strategy [55,56]. This individualized approach, as further described by Banovic et al., facilitates timely intervention if needed, while safely delaying surgery in low-risk individuals who remain truly asymptomatic [21]. Such studies suggest that a watchful waiting strategy, with strategized surveillance and patient-specific risk stratification, can be an effective approach in many patients with asymptomatic severe AS.

## 5. Early Intervention: A Lifesaving Approach?

On the other hand, emerging evidence continues to challenge the traditional ‘wait-for-symptoms’ paradigm in managing asymptomatic severe AS. Several recent investigations, including meta-analyses, randomized trials, and long-term follow-up studies, suggest that earlier surgical intervention may offer clinical benefits even before symptomatic progression occurs. Ullah and colleagues reported that early intervention was associated with a marked reduction in all-cause mortality (OR 0.24, [95% CI 0.13–0.45], *p* ≤ 0.00001) and cardiovascular mortality (OR 0.21, [95% CI 0.06–0.70], *p* = 0.01) compared to conservative management strategies, with a number needed to treat of four to prevent one all-cause mortality [57]. A meta-analysis by Yokoyama, Takagi, and Kuno found that early surgery nearly halved all-cause mortality (HR 0.49, [95% CI 0.36–0.68], *p* < 0.0001) and significantly lowered cardiovascular mortality (HR 0.42, [95% CI 0.22–0.82], *p* = 0.01), highlighting that the survival advantage extended to both severe and very severe forms of AS [58]. Similarly, a meta-analysis by Costa et al. further demonstrated that all-cause mortality (OR 0.40, [95% CI 0.35–0.45], *p* < 0.01), cardiovascular mortality (OR 0.33, [95% CI 0.19–0.56], *p* < 0.01), and heart failure hospitalization (OR 0.19, [95% CI 0.10–0.39], *p* < 0.01) were significantly lower for the early AVR group compared to watchful waiting [59]. In addition, Ahmad et al. demonstrated in their meta-analysis of randomized controlled trials that early SAVR led to a 55% reduction in all-cause mortality (HR 0.45, [95% CI 0.24–0.85], *p* = 0.014) and a 79% reduction in heart failure hospitalizations (HR 0.21, [95% CI 0.05–0.96], *p* = 0.044) [60]. These findings not only highlight the survival and morbidity benefits of early SAVR in patients with severe asymptomatic AS but also challenge current treatment standards.

Randomized trials have also provided a framework for understanding these benefits. The AVATAR trial, as reported by Banovic et al., demonstrated that patients undergoing early surgical intervention experienced significantly fewer composite events—encompassing all-cause death, myocardial infarction, stroke, or heart failure hospitalizations—than those managed with watchful waiting [HR 0.42, 95% CI 0.24–0.73, *p* = 0.002]. After a median follow-up of 63 months, the rate of all-cause death was significantly lower in the early surgery group (HR 0.44, [95% CI 0.23–0.85], *p* = 0.012), as was the rate of heart failure hospitalizations (HR 0.21, [95% CI 0.06–0.73], *p* = 0.007) [9]. This trial’s extended follow-up data confirm that the survival benefits and event reduction persist over time. Kang and colleagues’ study further supported these conclusions, showing that a primary endpoint event (i.e., operative mortality or death from cardiovascular causes) occurred in only 1% of patients in the early-surgery group compared to 15% in the conservative-care group who only had surgery when symptomatic (HR 0.09, [95% CI 0.01–0.67], *p* = 0.003). Death from any cause was also significantly lower in the early-surgery group (7%) compared to the conservative-care group (21%) (HR 0.33, [95% CI 0.12–0.90]), with the cumulative incidence of sudden death in the conservative-care group reaching 14% at 8 years [61]. These randomized trials challenge the notion that asymptomatic status itself warrants delay, demonstrating that, at least for high-risk patients, intervention before clinical deterioration is beneficial.

## 6. Recent Clinical Trials

### 6.1. The EVOLVED Randomized Clinical Trial

The EVOLVED Randomized Clinical Trial was a parallel-group, multicenter, prospective trial investigating whether early aortic valve intervention can improve clinical outcomes in patients with asymptomatic severe AS and myocardial fibrosis [62]. The trial was conducted between August 2017 and October 2022 at 24 cardiac centers across the United Kingdom and Australia.

This study included patients aged 18 years and older with severe asymptomatic AS, defined as an aortic valve peak velocity ≥4 m/s or ≥3.5 m/s with an indexed AVA < 0.6 cm^2^/m^2^. The presence of myocardial fibrosis was independently assessed by the evaluation of midwall late gadolinium enhancement. Exclusion criteria included symptomatic AS, LVEF < 50%, severe aortic or mitral regurgitation, advanced kidney disease (GFR < 30 mL/min/1.73 m^2^), MRI contraindications, or unsuitability for surgery or TAVR. Patients in the intervention group underwent either SAVR or TAVR, as determined by the local heart valve team. Those assigned to clinical surveillance were referred for AVR if deemed appropriate by their treating physician and local heart valve team.

The study’s primary endpoints included a composite of all-cause mortality and unplanned AS-related hospitalization. Secondary endpoints included the individual components of the composite primary endpoint, symptom burden (assessed by the New York Heart Association [NYHA] classification), development of left ventricular systolic dysfunction (LVEF < 50%), cardiovascular death, AS-related death, stroke, endocarditis, cardiac pacemaker implantation or resynchronization device placement, and post-operative complications within 30 days.

A total of 224 asymptomatic patients were included in the study, with 113 receiving early intervention and 111 assigned to the clinical surveillance group. There were no significant differences between the early intervention group and the clinical surveillance group regarding the primary endpoint (18% vs. 23%, HR, 0.79 [95% CI, 0.44–1.43], *p* = 0.44), and overall mortality (14% vs. 13%, HR, 1.22 [95% CI, 0.59–2.51]). On the other hand, there were significant benefits for the early intervention group regarding unplanned AS-related hospitalization (6% vs. 17%, HR, 0.37 [95% CI, 0.16–0.88]) and 1-year development of new NYHA class II–IV symptoms (20% vs. 38%, OR, 0.37 [95% CI, 0.20–0.70]).

In this trial, authors tried to prove the hypothesis that increased-risk populations with evidence of myocardial fibrosis—potentially representing decompensating LV function due to severe valve dysfunction—would be the most to gain from early intervention. However, this impact was not demonstrated in the primary outcomes.

### 6.2. The EARLY TAVR Trial

In comparison, the EARLY TAVR Trial was a prospective, randomized-controlled, multicenter trial that compared TAVR (SAPIEN3 or SAPIEN3 Ultra, Edwards, Lifesciences) and clinical surveillance among patients with asymptomatic severe AS from 75 sites in the United States and Canada [63]. Asymptomatic patients older than 65 years with anatomy suitable for transfemoral TAVR were included. Exclusion criteria included a Society of Thoracic Surgeons Predicted Risk of Mortality (STS-PROM) score > 10%, LVEF ≤ 50%, or any other class I indications for AVR. Asymptomatic status was determined by a negative stress test or a detailed physician assessment of the medical history.

The primary endpoint of the study was a composite of death from any cause, stroke, and unplanned hospitalization for CV causes. Secondary endpoints included a favorable outcome at 2 years, defined as being alive with a Kansas City Cardiomyopathy Questionnaire (KCCQ) score of at least 75 that did not decrease more than 10 points from baseline. Other secondary endpoints included integrated measures of LV and left atrial (LA) health at 2 years, change in LVEF from baseline in 2 years, new-onset atrial fibrillation, and a composite of death or disabling stroke.

A total of 901 patients were randomized, with 455 undergoing TAVR and 446 assigned to clinical surveillance. The composite primary endpoint, including death from any cause, stroke, or unplanned hospitalization for CV causes, occurred in 122 patients (26.8%) in the TAVR group compared with 202 patients (45.3%) in the clinical surveillance group (HR, 0.50, [95% CI, 0.40–0.63], *p* < 0.001). Moreover, this was mainly driven by a benefit in the TAVR group regarding unplanned hospitalization (20.9% vs. 41.7%, HR, 0.43, [95% CI, 0.33–0.55]), with no significant differences in death from any cause (8.4 vs. 9.2 HR, 0.93, [95% CI,0.60–1.44]), and stroke (4.2% vs. 6.7%, HR, 0.62, [95% CI, 0.35–1.10]).

Regarding the secondary endpoint, a favorable outcome at 2 years occurred in 86.6% of patients in the TAVR group and 68.0% of patients in the clinical surveillance group (*p* < 0.001). Integrated LV and LA measures 2 years occurred in 48.1% and 35.9% of patients, respectively (*p* < 0.001). Among the 446 patients randomly assigned to clinical surveillance, 388 (87.0%) underwent AVR during follow-up, with the median time from randomization to conversion to AVR being 11.1 months (IQR, 5.0–19.7).

In contrast to The EVOLVED Randomized trial, The EARLY TAVR trial demonstrated that early TAVR was superior to clinical surveillance regarding the primary composite end point and secondary endpoints. The percentage of mortality appeared to be similar in both groups but was lower than in previous trials, which can be explained by the less invasive nature of TAVR compared to surgery.

Prospective studies and ongoing trials continue to refine patient selection criteria and optimal timing. Richardson et al. have laid the groundwork for future investigations by detailing trial designs that specifically address the question of early AVR timing in asymptomatic severe AS [64]. By continuing to explore the interplay between clinical, echocardiographic, and biomarker-based risk stratification, such studies aim to delineate which patients would have the most benefit from early intervention and to what extent these advantages outweigh operative risks and potential prosthesis-related complications over the long term.

## 7. Future Perspectives

We are living in a world of unprecedented transformation, driven by the rapid advancements of Artificial Intelligence (AI). AI is no longer a futuristic concept; it is a reality with the potential to revolutionize numerous fields, including healthcare. In the context of asymptomatic aortic stenosis, AI can be a powerful tool to improve patient care. AI can analyze vast datasets of patient information including but not limited to, imaging, wearable, clinical, and genomic data. By analyzing this complex data, AI may develop more accurate risk stratification models to personalize treatment plans and optimize the timing of intervention. For example, the PROGRESSA study put yearly clinical and echocardiographic data from 303 patients through various machine and deep learning algorithms to identify those at risk for rapid clinical or hemodynamic progression of AS. Several machine learning models showed a higher area under the curve compared to the traditional clinical model -which was designed to reflect current clinical practice accuracy and features associated with faster progression of AS. These models included LightGBM (AUC = 0.85, 95% CI: 0.83–0.86), XGBoost (AUC = 0.84, 95% CI: 0.83–0.85]), GRU (AUC = 0.81 [95% CI: 0.79–0.82]), and LSTM (AUC = 0.81 [95% CI: 0.79–0.82]). AI tools and software that automatically quantify aortic valve composition, consistently outperformed standard clinical models in predicting outcomes in AS progression, promising a futuristic approach to risk stratifying AS in asymptomatic patients [65].

## 8. Conclusion

Aortic stenosis is the most prevalent valvular disease, often leading to serious morbidities and adverse outcomes. While most patients are asymptomatic at the time of initial diagnosis many face the risk of progressing to severe disease. Timely intervention and careful consideration are crucial to effectively manage these asymptomatic patients. Evaluation through exercise testing, echocardiography, CT, MRI cardiac biomarkers, and the evolution of AI to help contextualize vast amounts of data may all help to refine those perceived as high-risk, to experience rapid progression or adverse outcomes. Clinicians must remain vigilant, as the strategy of watchful waiting in those with asymptomatic aortic stenosis patients is evolving, with emerging evidence suggesting that early intervention may soon become the new standard for those at high risk of rapid progression or adverse outcomes.
